# Space-Time Block Coded Cooperative MIMO Systems

**DOI:** 10.3390/s21010109

**Published:** 2020-12-26

**Authors:** Han Hai, Caiyan Li, Jun Li, Yuyang Peng, Jia Hou, Xue-Qin Jiang

**Affiliations:** 1 Engineering Research Center of Digitized Textile & Apparel Technology, College of Information Science and Technology, Donghua University, Shanghai 201620, China; hhai@dhu.edu.cn (H.H.); cyli@mail.dhu.edu.cn (C.L.); 2 Research Center of Intelligent Communication Engineering, School of Electronics and Communication Engineering, Guangzhou University, Guangzhou 510006, China; lijun52018@gzhu.edu.cn; 3 Faculty of Information Technology, Macau University of Science and Technology, Macau 999078, China; yypeng@must.edu.mo; 4 College of School of Electronic and Information Engineering, Soochow University, Suzhou 215006, China; houjia@suda.edu.cn

**Keywords:** MIMO, cooperative MIMO, STBC

## Abstract

The main objective of a Cooperative Multiple-Input Multiple-Output (CMIMO) system is to improve network throughput and network coverage and save energy. By grouping wireless devices as virtual multi-antenna nodes, it can thus simulate the functions of multi-antenna systems. A Space-Time Block Code (STBC) was proposed to utilize the spatial diversity of MIMO systems to improve the diversity gain and coding gain. In this paper, we proposed a cooperative strategy based on STBC and CMIMO, which is referred to as Space-Time Block Coded Cooperative Multiple-Input Multiple-Output (STBC-CMIMO) to inherit the advantages from both STBC and CMIMO. The theoretical performance analysis for the proposed STBC-CMIMO is presented. The performance advantages of the STBC-CMIMO are also shown by simulations. In the simulations, it is demonstrated that STBC-CMIMO can obtain significant performance compared with the existing CMIMO system.

## 1. Introduction

The Multiple-Input Multiple-Output (MIMO) system has been a key technology for modern wireless systems in recent years. It offers better error performance and higher data rates [1,2] than that of conventional communication systems. However, high Inter-Channel Interference (ICI) in MIMO systems requires a complex receiver algorithm, which leads to high complexity [3].

The channel of wireless communication has broadcast characteristics. Cooperative communication [4,5,6] uses this feature for information transmission. The generation of cooperative communication is inspired by the concept of relay communication. In 1979, the author of [7] first used the viewpoint of information theory to study the classical three node relay cooperation model of Source (S), Relay (R) and Destination (D). The development of cooperative communication technology has attracted wide attention since Sendonaris proposed the concept of cooperative diversity in 1998 [8]. In the process of signal transmission, S transmits information to D. In addition, R can also receive the signal transmitted from S. R retransmits the signal to D after decoding and recoding. D receives the same signal from S and R. In this virtual MIMO system, users participating in cooperation can forward information to each other, and the receiver can receive the same information from different paths, so as to obtain diversity gain. The commonly used communication protocols at R are Amplification-and-Forward (AF) and Decode-and-Forward (DF) proposed by Laneman [9] and Coded Cooperation (CC) proposed by Hunter [10]. As an important means to combat channel fading, cooperative communication has been widely concerned by scholars at home and abroad in recent years. Cooperative MIMO (CMIMO), also referred to as distributed, virtual, or networked MIMO, is one type of cooperative communication. In CMIMO, there are several nodes, and each node is equipped with multiple antennas. They cooperate to emulate a multi-antenna node, also known as a virtual antenna array.

In order to solve these problems without loss of the Bit Error Rate (BER) performance and transmission rate, Spatial Modulation (SM) [11] is proposed. In SM, only one transmit antenna is active at any time slot [12]. The information symbols are not only transmitted by the activated antenna, but also the index of the activated antenna. Therefore, ICI at the receiver can be completely avoided, since only one transmit antenna will be active at each time instant while the other antennas will keep silent. SM has recently been considered as a promising technique for the next generation wireless communication systems [13,14,15]. As a result, the transmission rate of SM is limited when the number of transmit antennas is large. Hence, a Generalized Spatial Modulation (GSM) system [16] is proposed to increase the transmission rate. Not like SM, GSM uses multiple activated transmit antennas during transmission. However, all the activated transmit antennas the same information symbols. In order to further exploit high spectral efficiency, Multiple-Active Spatial Modulation (MA-SM) [17] is proposed. In MA-SM, different information symbols are transmitted by the different activated transmit antennas during one time slot, which can further increase the transmission rate of the system.

Space-Time Block Code (STBC) [18,19,20,21] technology has been continuously of concern since the mid-1990s because of its unique advantages, and has been widely used in many communication scenarios. Space time code (STC) technology takes into account the coding scheme of the wireless channel, the size of spatial diversity gain, the complexity of detection and demodulation algorithm and other factors that affect the system performance, and integrates them through related technologies. Therefore, the system capacity, transmission efficiency and reliability potential in the MIMO system are developed to the maximum extent. In addition, various modulation techniques and modulation recognition techniques have been proposed. The common technologies include Blind Modulation Identification (BMI), and Golden Angle Modulation (GAM). Compared with the traditional modulation technology, these new technologies can extend new research fields in the research of the MIMO system, so they have been widely concerned by scholars.

Recently, several wireless communication systems based on STBC and SM techniques have been studied for CMIMO in [22,23,24,25,26,27,28]. Specifically, a space-time mapping for equiprobable antenna activation is designed for SM in [22] to achieve better performance with a marginal increment in detection complexity. In [23], the authors proposed novel noncoherent massive space-time block codes for uplink relay communications. The authors of [24] proposed a switched relaying framework for CMIMO systems, which uses a novel relay selection protocol to select the best links among several relay nodes. In [25], a new scheme is proposed to extend the size of a spatial constellation diagram for a spatial modulation system to improve the spectral efficiency. Authors of [26] investigated a secure transmission by adding artificial noise to spatial modulation system, which can improve the secrecy capacity. Then, low-complexity differential SM schemes for hundreds of antennas are studies in [27], which can decrease the complexity for the detector. In [28], the authors studied the antenna selection for an offset SM system based on grouped Euclidean distance and channel coefficient, which provides a flexible tradeoff between computational complexity and system performance.

In this paper, a novel MIMO communication system is proposed, which is referred to as STBC-CMIMO. In STBC-CMIMO, data is transmitted by an STBC matrix [2], which is coded according to GSM encoding rules. Furthermore, DF protocol is utilized in the proposed STBC-CMIMO. At the receiver side, the index of the activated receive antenna and the transmitted symbol can be estimated by the Maximum Likelihood (ML) detector. Therefore, the proposed STBC-CMIMO system can take advantages of the CMIMO, GSM and STBC.

In detail, the contributions of this letter can be summarized as:

- A novel wireless communication system, called the STBC-CMIMO system, is proposed. Information bits in STBC-CMIMO are mapped into the STBC matrix by GSM encoding rules and then transmitted via a CMIMO network.
- An analytical upper bound of the BER performance for STBC-CMIMO is given and analytical results are validated through Monte Carlo simulation results.
- Compared to the conventional CMIMO system, the proposed STBC-CMIMO system can achieve better BER performance.

The remainder of this paper is organized as follows. Section 2 presents the system model of STBC-CMIMO. In Section 3, we analyze the BER upper bound of the proposed STBC-CMIMO. Simulation results are given in Section 4. Finally, Section 5 concludes the paper.

*Notation:* Boldface upper case letters represent matrices and boldface lower case letters represent vectors, respectively. ∥·∥ and ${\left(\cdot \right)}^{T}$, ${\left(\cdot \right)}^{*}$ represent Euclidean norm, transposition and complex conjugation, respectively. $C^{m\times n}$ stands for the complex space of size m×n dimensions. ⌊·⌋ indicates flooring operator and ·· represents the binomial coefficient. E[·] evaluates the expectation with respect to all random variables within the bracket. Pr{·} denotes the probability of an event.

## 2. System Model

**Figure 1:**
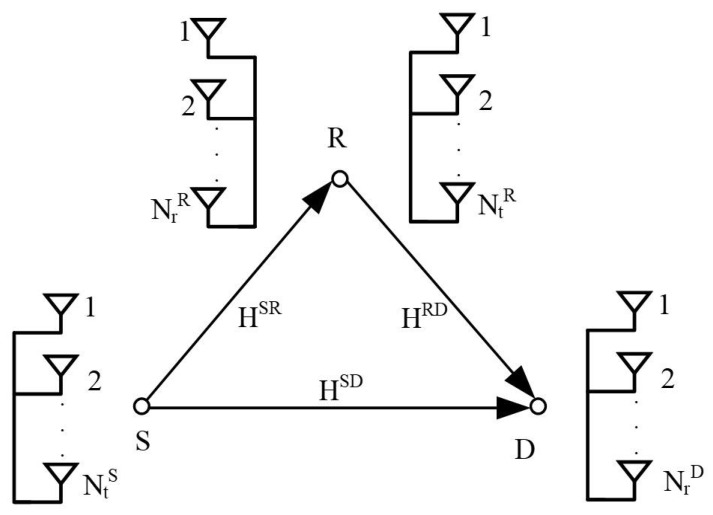
System model.

We consider a cooperative communication system consisting of a single relay. In addition, DF relaying is used in the proposed STBC-CMIMO system. The STBC-CMIMO model is shown in Figure 1. In the STBC-CMIMO system, *S* and *R* have $N_{t}^{S}$ and $N_{t}^{R}$ transmit antennas and *R* and *D* have $N_{t}^{R}$ and $N_{t}^{D}$ receive antennas. The channel matrix between *S* and *R*, *S* and *D*, *R* and *D* are denoted as $H^{SR}\in C^{N_{r}^{R}\times N_{t}^{S}}$, $H^{SD}\in C^{N_{r}^{D}\times N_{t}^{S}}$, and $H^{RD}\in C^{N_{r}^{D}\times N_{t}^{R}}$. $h_{i,j}^{SR},h_{i,j}^{SD},h_{i,j}^{RD}∼\mathrm{CN}\left(0,\sigma^{2}\right)$ representing the element of *i*-th row and the *j*-th column of $H^{SR}$, $H^{SD}$ and $H^{RD}$, respectively, where $\sigma^{2}$ represents $\sigma_{SR}^{2}$, $\sigma_{SD}^{2}$ and $\sigma_{RD}^{2}$, respectively. In the STBC-CMIMO system, information bits will be divided into several blocks. Each block is with the length of $\eta =\eta_{l}+\eta_{s}$, where ηl=⌊Nr2⌋ and $\eta_{s}=2\log_{2}M$. Therefore, for each block, $\eta_{l}$ bits can be conveyed by an index of activated receive antennas Φ=(i,j), and $\eta_{s}$ bits can be conveyed by the STBC matrix [2]

(1) $$\begin{array}{c} S=\left(\begin{array}{cc} x_{1} & x_{2}\\ -x_{2}^{*} & x_{1}^{*} \end{array}\right), \end{array}$$

where $x_{1}$ and $x_{2}$ are two complex information symbols drawn from *M*-PSK or *M*-QAM constellation transmitted to two activated receive antennas in two time slots. The transmission matrix $X\in C^{N_{r}\times 2}$ can be formulated as

(2) $$\begin{array}{c} X={\left(\begin{array}{ccccc} \ldots & x_{1} & \ldots & x_{2} & \ldots \\ \ldots & \underset{i-\mathrm{th}\;\mathrm{position}}{\underbrace{-x_{2}^{*}}} & \ldots & \underset{j-\mathrm{th}\;\mathrm{position}}{\underbrace{x_{1}^{*}}} & \ldots \end{array}\right)}^{T} \end{array}$$

Examples of transmission matrices X with $N_{t}=4$ and $N_{r}=8$ are provided in Table 1.

In the first time slot, X is transmitted from *S*. The receive signal matrixes at *R* and *D* can be expressed as

(3) $$\begin{array}{c} Y^{SR}=H^{SR}X+N^{SR}, \end{array}$$

(4) $$\begin{array}{c} Y^{SD}=H^{SD}X+N^{SD}, \end{array}$$

where $N^{SR}\in C^{N_{r}^{R}\times 1}$ and $N^{SD}\in C^{N_{r}^{D}\times 1}$ are the additive complex Gaussian noise matrices, the entries of which follow CN(0,σ).

We further assume that the channel information is known at *R*. An optimal Maximum Likelihood (ML) detector must make an exhaustive search over all possible transmission matrices. Therefore, in the second time slot, the detector can be written as

(5) $$\begin{array}{c} \left(\tilde{\Phi },\tilde{S}\right)=\arg\min{∥Y^{SR}-H^{SR}X∥}^{2}. \end{array}$$

$\tilde{\Phi }$ and $\tilde{S}$ are re-encoded into $\tilde{X}$, which is transmitted from *R* to *D*. The mapping rule is shown in Table 1. The receive signal matrix at *D* can be expressed as

(6) $$\begin{array}{c} Y^{RD}=H^{RD}\tilde{X}+N^{RD}, \end{array}$$

where $N^{RD}\in C^{N_{r}^{D}\times 1}$ is the additive complex Gaussian noise matrix, the entries of which follow CN(0,σ).

The optimal ML detector at *D* is formed as

(7) $$\begin{array}{c} \left(\overset{⌢}{\Phi },\overset{⌢}{S}\right)=\arg\min\left({∥Y^{SD}-H^{SD}X∥}^{2}+{∥Y^{RD}-H^{RD}X∥}^{2}\right). \end{array}$$

## 3. Theoretical Performance

In the following, we provide the theoretical performance of the proposed STBC-CMIMO system. The upper bound on the Average Pairwise Error Probability (APEP) of the ML detection for the proposed STBC-CMIMO system at *D* can be expressed as

(8) $$\begin{array}{c} P_{D}^{DF}\left(X\to \overset{⌢}{X}\right)\leq P_{R}\left(X\right)P_{D}\left(X\to \overset{⌢}{X}\left|R:X\right.\right)+\sum P_{R}\left(X\to \tilde{X}\right)P_{D}\left(X\to \overset{⌢}{X}\left|R:\tilde{X}\right.\right), \end{array}$$

where $P_{R}\left(X\to \tilde{X}\right)$ is the APEP when X is erroneously detected as $\tilde{X}$ at *R*. $P_{R}\left(X\right)$ is the probability of correct detection at *R*. $P_{D}(X\to \overset{⌢}{X}\left|R:X\right)$ is the APEP of *D* when correct detection is done at *R*. $P_{D}(X\to \overset{⌢}{X}\left|R:\tilde{X}\right)$ is the APEP of *D* when erroneous detection is done at *R*.

$P_{D}\left(X\to \overset{⌢}{X}\left|R:\tilde{X}\right.\right)$ can be expressed as

(9) $$\begin{array}{cc} \; & P_{D}\left(X\to \overset{⌢}{X}\left|R:\tilde{X}\right.\right)=E\{\mathrm{Pr}\{{∥Y^{SD}-H^{SD}X∥}^{2}+{∥Y^{RD}-H^{RD}X∥}^{2}\\ \; & \geq {∥Y^{SD}-H^{SD}\overset{⌢}{X}∥}^{2}+{∥Y^{RD}-H^{RD}\overset{⌢}{X}∥}^{2}\left|H^{SD},\right.H^{RD}\}\}\\ \; & =E\{\mathrm{Pr}\{{∥\left(H^{SD}X+N^{SD}\right)-H^{SD}X∥}^{2}\\ \; & +{∥\left(H^{RD}\tilde{X}+N^{RD}\right)-H^{RD}X∥}^{2}\\ \; & \geq {∥\left(H^{SD}X+N^{SD}\right)-H^{SD}\overset{⌢}{X}∥}^{2}\\ \; & +{∥H^{RD}\tilde{X}+N^{RD}∥}^{2}\left|H^{SD},\right.H^{RD}\}\}, \end{array}$$

which can be simplified to

(10) $$\begin{array}{cc} \; & P_{D}\left(X\to \overset{⌢}{X}\left|R:\tilde{X}\right.\right)\\ = & E\left\{Q\left(\frac{{∥H^{SD}\left(X-\overset{⌢}{X}\right)∥}^{2}+{∥H^{RD}\left(\tilde{X}-\overset{⌢}{X}\right)∥}^{2}-{∥H^{RD}\left(\tilde{X}-X\right)∥}^{2}}{\sqrt{2N_{0}\left({∥H^{SD}\left(X-\overset{⌢}{X}\right)∥}^{2}+{∥H^{RD}\left(\tilde{X}-\overset{⌢}{X}\right)∥}^{2}+{∥H^{RD}\left(\tilde{X}-X\right)∥}^{2}\right)}}\right)\right\}. \end{array}$$

Equation (10) is greater than (9) because of the strict decreasing of the $Q\left(\cdot \right)$ function. In addition, when $N_{0}\to 0$, (10) can be further expressed as [29]

(11) $$\begin{array}{c} P_{D}\left(X\to \overset{⌢}{X}\left|R:\tilde{X}\right.\right)≃\mathrm{Pr}\left({∥H^{SD}\left(X-\overset{⌢}{X}\right)∥}^{2}<{∥H^{RD}\left(X-\overset{⌢}{X}\right)∥}^{2}\left|H^{SD},\right.H^{RD}\right). \end{array}$$

It can be calculated that (11) is about equal to 0.5, which means that the ABEP of the proposed STBC-CMIMO system can be determined by $\tilde{X}=\overset{⌢}{X}$. Therefore, (8) is further expressed as

(12) $$\begin{array}{c} P_{D}^{DF}\left(X\to \overset{⌢}{X}\right)≃P_{R}\left(X\right)P_{D}\left(X\to \overset{⌢}{X}\left|R:X\right.\right)+\sum P_{R}\left(X\to \tilde{X}\right)P_{D}\left(X\to \overset{⌢}{X}\left|R:\tilde{X}\right.\right), \end{array}$$

where $P_{R}\left(X\to \tilde{X}\right)$ is expressed as

(13) $$\begin{array}{c} P_{R}\left(X\to \tilde{X}\right)=E\left\{\mathrm{Pr}\left\{X\to \overset{⌢}{X}\left|H^{SR}\right.\right\}\right\}=E\left\{Q\left\{\sqrt{\frac{{∥H^{SR}\left(X-\overset{⌢}{X}\right)∥}^{2}}{2N_{0}}}\right\}\right\}. \end{array}$$

$P_{R}\left(X\right)$ is determined by $\tilde{X}=\overset{⌢}{X}$, too. It can be approximated as $P_{R}\left(X\right)≃\left(1-P_{R}\left(X\to \tilde{X}\right)\right)$.

$\tilde{\Phi }=\Phi$ and $\tilde{S}=S$, i.e., $\tilde{X}=X$, hold in $P_{D}\left(X\to \overset{⌢}{X}\left|R:\tilde{X}\right.\right)$. So $P_{D}\left(X\to \overset{⌢}{X}\left|R:\tilde{X}\right.\right)$ can be simplified as

(14) $$\begin{array}{c} P_{D}\left(X\to \overset{⌢}{X}\left|R:\tilde{X}\right.\right)=E\left\{Q\left(\frac{{∥H^{SD}\left(X-\overset{⌢}{X}\right)∥}^{2}+{∥H^{RD}\left(X-\overset{⌢}{X}\right)∥}^{2}}{2N_{0}}\right)\right\}. \end{array}$$

To calculate the APEP, the Probability Density Function (pdf) of the random variable in the $Q\left(\cdot \right)$ function has to be calculated. $f_{\gamma^{SR}}\left(\gamma \right)$ is the pdf of $\gamma^{SR}≜\left(\rho /2\right){∥H^{SR}\left(X-\overset{⌢}{X}\right)∥}^{2}$. The APEP of *R* can be expressed as

(15) $$\begin{array}{c} P_{R}\left(X\to \tilde{X}\right)=\int_{0}^{\infty }Q\left(\sqrt{\gamma^{SR}}\right)f_{\gamma^{SR}}\left(\gamma \right)d\gamma . \end{array}$$

It is calculated with Craig’s formula [30]. Equation (15) is further expressed as

(16) $$\begin{array}{c} P_{R}\left(X\to \tilde{X}\right)=\frac{1}{\pi }\int_{0}^{\pi /2\;}M_{\gamma^{SR}}\left(-\frac{1}{2\sin^{2}\theta }\right)d\theta , \end{array}$$

where $M_{\gamma^{SR}}\left(s\right)$ is the moment generating function of $\gamma^{SR}$. $\gamma^{SR}$ obeys the distribution of $\gamma (N_{r}^{R},\left(\rho \lambda_{x}\sigma_{SR}^{2}\right))$, where $\lambda_{x}$ can be expressed as

(17) $$\begin{array}{c} \lambda_{x}=\left\{\begin{array}{ccc} |S-\overset{⌢}{X}| & \mathrm{if} & \Phi =\overset{⌢}{\Phi }\\ {\left|S\right|}^{2}+{\left|\overset{⌢}{X}\right|}^{2} & \mathrm{if} & \Phi \neq \overset{⌢}{\Phi } \end{array}.\right. \end{array}$$

$M_{\gamma^{SR}}\left(s\right)$ can be expressed as [30]

(18) $$\begin{array}{c} M_{\gamma^{SR}}\left(s\right)={\left(1-\frac{\rho \lambda_{x}\sigma_{SR}^{2}}{2}s\right)}^{-N_{r}^{R}}. \end{array}$$

Therefore, (16) is further expressed as

(19) PR(X→X˜)=121−μ∑j=0NrR−12jj1−μ24j,

where

(20) $$\begin{array}{c} \mu =\sqrt{\frac{\frac{\rho \lambda_{x}\sigma_{SR}^{2}}{4}}{\frac{\rho \lambda_{x}\sigma_{SR}^{2}}{4}+1}}. \end{array}$$

The same procedures can be used to calculate $P_{D}\left(X\to \overset{⌢}{X}\left|R:X\right.\right)$, which can be expressed as

(21) PD(X→X⌢R:X)=121−μ∑j=02NrD−12jj1−μ24j.

Therefore, the ABEP can be expressed as [31]

(22) $$\begin{array}{c} P_{b}^{DF}≃\frac{1}{N_{t}M\log_{2}\left(N_{t}M\right)}\sum_{X}\sum_{\begin{array}{c} \overset{⌢}{X}\\ \overset{⌢}{X}\neq X \end{array}}d\left(X\to \overset{⌢}{X}\right)P_{D}^{DF}\left(X\to \overset{⌢}{X}\right), \end{array}$$

where $d(X\to \overset{⌢}{X})$ denotes the number of bits in error between X and $\overset{⌢}{X}$.

When θ=π/2, we have

(23) $$\begin{array}{c} P_{R}\left(X\to \tilde{X}\right)\leq {\left(1+\frac{\rho \lambda_{x}\sigma_{SR}^{2}}{4}\right)}^{-N_{r}^{R}}, \end{array}$$

(24) $$\begin{array}{c} P_{D}\left(X\to \overset{⌢}{X}\left|R:X\right.\right)\leq {\left(1+\frac{\rho \lambda_{x}\sigma_{SD}^{2}}{4}\right)}^{-N_{r}^{D}}{\left(1+\frac{\rho \lambda_{x}\sigma_{RD}^{2}}{4}\right)}^{-N_{r}^{D}}. \end{array}$$

## 4. Simulation Result

In this section, we present the theoretical and simulation results for the BER performance of STBC-CMIMO. We also compare the BER performance of the proposed STBC-CMIMO system with conventional CMIMO. In addition, in order to obtain the same spectral efficiency, the number of transmit antennas at *S* is equal to *R*, i.e., $N_{t}^{S}=N_{t}^{R}$, and the modulation order of the two processes are fixed to be $M^{S}=M^{R}=M$.

**Figure 2:**
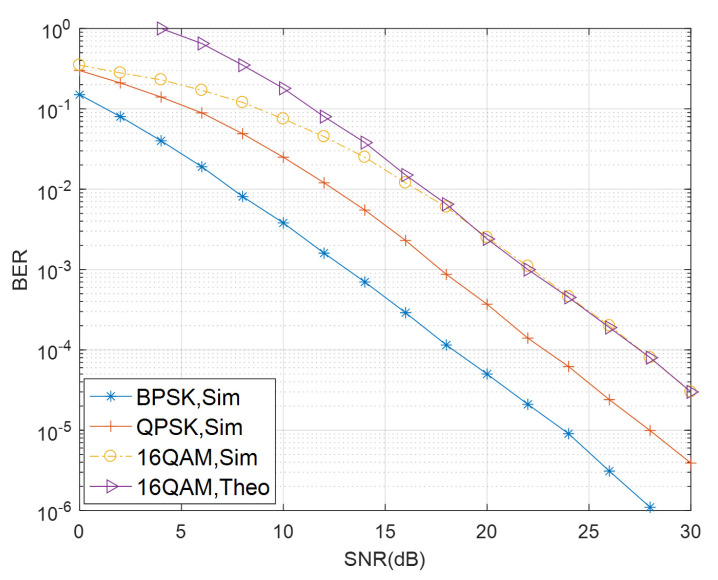
Performance comparison of the STBC-CMIMO system for $N_{t}^{S}=N_{t}^{R}=N_{r}^{R}=N_{r}^{D}=4$ with BPSK, QPSK and 16QAM.

Figure 2 investigates the BER performance of the proposed STBC-CMIMO system. *S* and *R* equip the same transmit antenna number $N_{t}^{S}=N_{t}^{R}=4$. *R* and *D* equip the same receive antenna number $N_{r}^{R}=N_{r}^{D}=4$. BPSK, QPSK and 16-QAM are adopted in the simulation, respectively. To evaluate the derived upper bound, the theoretical bounds of the STBC-CMIMO system for 16-QAM is also shown in Figure 2. It is observed that both analytical and simulation results predict that the BER performance improves as the number of receiver antennas is increased. It can also be seen that the deviation between simulated and analytical results for 16-QAM is almost negligible, especially in the high SNR region.

**Figure 3:**
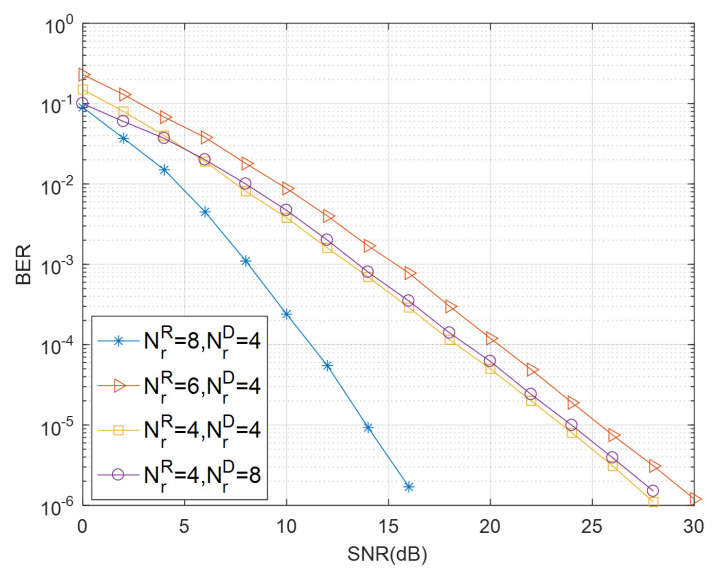
Bit Error Rate (BER) performance of a different number of receive antennas of STBC-CMIMO for $N_{t}^{S}=N_{t}^{R}=4$ with BPSK modulation.

Figure 3 illustrates the BER performance comparison of the proposed STBC-CMIMO for different receive antennas at *R* and *D*. The transmit antennas at *S* and *R* are fixed to be $N_{t}^{S}=N_{t}^{R}=4$. BPSK modulation is adopted in the simulation. It can be seen from Figure 3 that the number of the receive antennas at *R* has a significant impact on the performance of the STBC-CMIMO system, and the performance of the system improves when the number of receive antennas increases. However, the number of the receive antennas at *D* has a much smaller impact of the STBC-CMIMO system than that of *R*.

**Figure 4:**
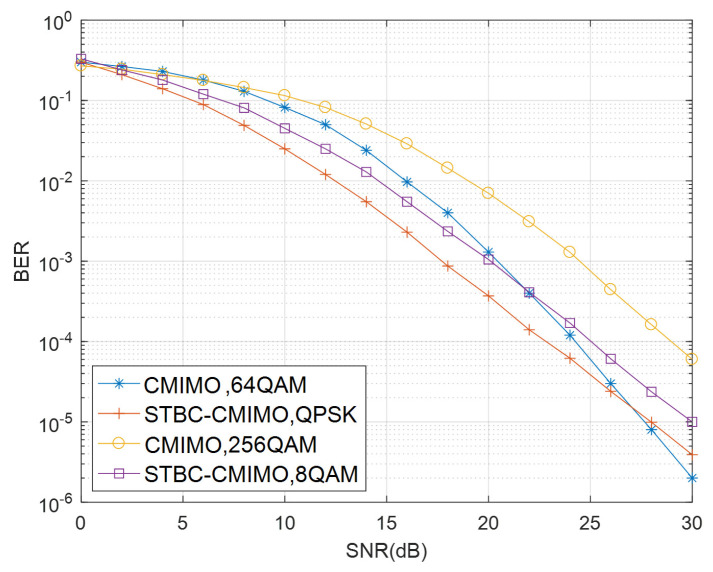
Performance comparison of STBC-CMIMO and conventional CMIMO at 6 and 8 bits/s/Hz spectral efficiencies.

We further compare the BER performance between STBC-CMIMO and conventional CMIMO systems with the transmission rates at 6 and 8 bits/s/Hz in Figure 4, respectively. The transmit antennas at *S* and *R* are fixed to be $N_{t}^{S}=N_{t}^{R}=4$ while the receive antennas at *R* and *D* are fixed to be $N_{r}^{R}=N_{r}^{D}=4$. We can see that when the transmission rate is 6 bits/s/Hz, the STBC-CMIMO system obtains a better performance than the conventional CMIMO system except in the high-SNR region. When the transmission rate is 8 bits/s/Hz, the STBC-CMIMO system has a significant performance gain compared to the conventional CMIMO system. The reason for this BER performance improvement can be explained by the fact that for the same spectral efficiency, the modulation order of conventional CMIMO is higher than that of the proposed STBC-MIMO, which leads to a better BER performance for STBC-CMIMO.

## 5. Conclusions

In this paper, we proposed a novel wireless communication system, which is referred to as STBC-CMIMO. The proposed STBC-CMIMO system utilizes both STBC and CMIMO to convey information. DF relaying is used in the proposed system. Furthermore, we also provided the upper bound on BER of the STBC-CMIMO system. This analytical result agreed well with the simulation result. Finally, we compared the BER performance of the STBC-CMIMO system to that of the conventional CMIMO system. Simulation results showed that the STBC-CMIMO system can achieve a better performance gain than the conventional CMIMO system.

## Figures and Tables

**Table 1:** Space-Time Block Code (STBC)-Cooperative Multiple-Input Multiple-Output (CMIMO) mapping table for $N_{t}=4$ and $N_{r}=8$.

Source Bits	Receive Antenna Combination Φ	Transmission Matrices
0000	(1,2)	$X={\left(\begin{array}{cccccccc} x_{1} & x_{2} & 0 & 0 & 0 & 0 & 0 & 0\\ -x_{2}^{*} & x_{1}^{*} & 0 & 0 & 0 & 0 & 0 & 0 \end{array}\right)}^{T}$
0001	(1,3)	$X={\left(\begin{array}{cccccccc} x_{1} & 0 & x_{2} & 0 & 0 & 0 & 0 & 0\\ -x_{2}^{*} & 0 & x_{1}^{*} & 0 & 0 & 0 & 0 & 0 \end{array}\right)}^{T}$
0010	(1,4)	$X={\left(\begin{array}{cccccccc} x_{1} & 0 & 0 & x_{2} & 0 & 0 & 0 & 0\\ -x_{2}^{*} & 0 & 0 & x_{1}^{*} & 0 & 0 & 0 & 0 \end{array}\right)}^{T}$
0011	(1,5)	$X={\left(\begin{array}{cccccccc} x_{1} & 0 & 0 & 0 & x_{2} & 0 & 0 & 0\\ -x_{2}^{*} & 0 & 0 & 0 & x_{1}^{*} & 0 & 0 & 0 \end{array}\right)}^{T}$
0100	(2,3)	$X={\left(\begin{array}{cccccccc} 0 & x_{1} & x_{2} & 0 & 0 & 0 & 0 & 0\\ 0 & -x_{2}^{*} & x_{1}^{*} & 0 & 0 & 0 & 0 & 0 \end{array}\right)}^{T}$
0101	(2,4)	$X={\left(\begin{array}{cccccccc} 0 & x_{1} & 0 & x_{2} & 0 & 0 & 0 & 0\\ 0 & -x_{2}^{*} & 0 & x_{1}^{*} & 0 & 0 & 0 & 0 \end{array}\right)}^{T}$
0110	(2,5)	$X={\left(\begin{array}{cccccccc} 0 & x_{1} & 0 & 0 & x_{2} & 0 & 0 & 0\\ 0 & -x_{2}^{*} & 0 & 0 & x_{1}^{*} & 0 & 0 & 0 \end{array}\right)}^{T}$
0111	(3,4)	$X={\left(\begin{array}{cccccccc} 0 & 0 & x_{1} & x_{2} & 0 & 0 & 0 & 0\\ 0 & 0 & -x_{2}^{*} & x_{1}^{*} & 0 & 0 & 0 & 0 \end{array}\right)}^{T}$
1000	(1,6)	$X={\left(\begin{array}{cccccccc} x_{1} & 0 & 0 & 0 & 0 & x_{2} & 0 & 0\\ -x_{2}^{*} & 0 & 0 & 0 & 0 & x_{1}^{*} & 0 & 0 \end{array}\right)}^{T}$
1001	(1,7)	$X={\left(\begin{array}{cccccccc} x_{1} & 0 & 0 & 0 & 0 & 0 & x_{2} & 0\\ -x_{2}^{*} & 0 & 0 & 0 & 0 & 0 & x_{1}^{*} & 0 \end{array}\right)}^{T}$
1010	(1,8)	$X={\left(\begin{array}{cccccccc} x_{1} & 0 & 0 & 0 & 0 & 0 & 0 & x_{2}\\ -x_{2}^{*} & 0 & 0 & 0 & 0 & 0 & 0 & x_{1}^{*} \end{array}\right)}^{T}$
1011	(2,6)	$X={\left(\begin{array}{cccccccc} 0 & x_{1} & 0 & 0 & 0 & x_{2} & 0 & 0\\ 0 & -x_{2}^{*} & 0 & 0 & 0 & x_{1}^{*} & 0 & 0 \end{array}\right)}^{T}$
1100	(2,7)	$X={\left(\begin{array}{cccccccc} 0 & x_{1} & 0 & 0 & 0 & 0 & x_{2} & 0\\ 0 & 0 & x_{1}^{*} & 0 & 0 & 0 & -x_{2}^{*} & 0 \end{array}\right)}^{T}$
1101	(2,8)	$X={\left(\begin{array}{cccccccc} 0 & x_{1} & 0 & 0 & 0 & 0 & 0 & x_{2}\\ 0 & -x_{2}^{*} & 0 & 0 & 0 & 0 & 0 & x_{1}^{*} \end{array}\right)}^{T}$
1110	(3,5)	$X={\left(\begin{array}{cccccccc} 0 & 0 & x_{1} & 0 & x_{2} & 0 & 0 & 0\\ 0 & 0 & -x_{2}^{*} & 0 & x_{1}^{*} & 0 & 0 & 0 \end{array}\right)}^{T}$
1111	(3,6)	$X={\left(\begin{array}{cccccccc} 0 & 0 & x_{1} & 0 & 0 & x_{2} & 0 & 0\\ 0 & 0 & -x_{2}^{*} & 0 & 0x_{1}^{*} & 0 & 0 \end{array}\right)}^{T}$

## Data Availability

Data sharing not applicable.
